# Oxygenation improvement and duration of prone positioning are associated with ICU mortality in mechanically ventilated COVID-19 patients

**DOI:** 10.1186/s13613-025-01438-y

**Published:** 2025-01-28

**Authors:** Silvia De Rosa, Nicolò Sella, Giacomo Bellani, Giuseppe Foti, Andrea Cortegiani, Giulia Lorenzoni, Dario Gregori, Annalisa Boscolo, Lucia Cattin, Muhammed Elhadi, Giorgio Fullin, Eugenio Garofalo, Leonardo Gottin, Alberto Grassetto, Salvatore Maurizio Maggiore, Elena Momesso, Mario Peta, Daniele Poole, Roberto Rona, Ivo Tiberio, Andrea Zanoletti, Emanuele Rezoagli, Paolo Navalesi, for the SIAARTI Study Group, Marco Abastanotti, Mohamed Abdel-Maboud Abdel-Maboud, Abdelfatah Abdellateef Abdelmageed, Eissa Abdullah, Ahmed Mohammed Abodina, Aymen Abuelyamen, Abdurraouf Abusalama, Tareg Abdalla Abuzaid, Romina Aceto, Stefano Addesa, Daniela Alampi, Amer Aldhalia, Risoli Alessio, Maytham Al-juaifari, Raja Ahmed Alqandouz, Mohammed Al-Sadawi, Marco Anderloni, Cannone Andrea, Enrico Andriolo, Botto Anna, Benvenuto Antonini, Gian Marco Anzellotti, Matteo Aritzu, Ahmed K. Awad, Flavio Badii, Hibah Bileid Bakeer, Aziza Bakri, Andrea Ballin, Massimo Barattini, Mattia Barotti, Mara Bassi, Mattia Bellandi, Marzia Bellin, Agrippino Bellissima, Annalisa Benini, Francesco Berruto, Giacomo Berta, Marco Berti, Emanuela Biagioni, Eugenio Biamonte, Giacomo Bianchetti, Andrea Bianchin, Manuela Biasetto, Muhannud Binnawara, Maria Bisi, Maria Maddalena Bitondo, Nicoletta Boffa, Michela Bombino, Benedetta Bonazzi, Elisa Bonetta, Elisa Boni, Sara Borga, Vincenzo Bosco, Gloria Boscolo, Veronica Bozzon, Luca Brazzi, Alessandro Bristot, Niccolò Brumana, Andrea Bruni, Chiara Bruscagnin, Stefano Busani, Guido Bussone, Pietro Caironi, Tiffany Calocero, Matteo Campagnolo, Claudine Canepa, Riccardo Carlon, Alfonso Carrara, Antonio Castelli, Giulia Catalisano, Martina Cavinato, Francesca Ceccaroni, Maurizio Cecconi, Martina Cedrone, Marcello Ceola Graziadei, Matteo Cesana, Nicola Cilloni, Enrico Colombo, Riccardo Colombo, Irene Coloretti, Sabrina Congedi, Angela Corea, Nicola Cornacchia, Silvia Corrado, Valentina Cricca, Chiara Crivellari, Wanda Cursio, Ernesto Dalla Mora, Lorenzo Dall’Ara, Vinicio Danzi, Luca Davicco, Alessandro De Cassai, Alessandro Devigili, Salvatore Di Blasi, Antonino Di Fede, Antonia Di Giannantonio, Pierluigi Di Giannatale, Luna Di Matteo, Giovanni Di Noto, Luca Di Tizio, Katia Donadello, Chiara Donatelli, Deyaa Elden Elbakheet, Gallo Elisa, Moataz Maher Emara, Haneen Esaadi, Mirca Fabbris, Massimo Ferluga, Luigi Ferrante, Francesco Filippone, Francesco Filippone, Elena Finotto, Ilaria Fior, Edoardo Forin, Tommaso Fossali, Rosa Fracchia, Salvo Francesco, Alessandra Gioia Francesconi, Nicola Franchetti, Sara Frisella, Regina Frontera, Roberto Fumagalli, Elisa Furlani, Lorenzo Gamberini, Elisa Gamberini, Emiliano Gamberini, Leonardo Gandolfi, Bernardetta Ganzerla, Silvia Gasperi, Ilenia Gatto, Federico Geraldini, Monica Geremia, Marco Giani, Massimo Girardis, Lorenzo Giuntoli, Ilaria Godi, Gianlorenzo Golino, Beatrice Gottardi, Antonio Grande, Massimiliano Greco, Daniela Guerra, Amedeo Guzzardella, Abdurrahman Haddud, Hashim Talib Hashim, Aliae A. R. Mohamed Hussein, Blangetti Ilaria, Teresa Iob, Giovanni Carmine Iovino, Mariachiara Ippolito, Stefano Isgrò, Cristina Jovinelli, Abdulmuhaymin Khalleefah, Ali Abdulnasir Kredan, Riccardo La Rosa, Claudio La Spisa, Luca Landolfi, Thomas Langer, Annalisa Lerose, Federico Linassi, Jacopo Lion, Giulia Lo Scrudato, Antonella Lombardo, Federico Longhini, Marta Lubian, Giulia Luccarelli, Alberto Lucchini, Aurora Magliocca, Giorgio Maiorelli, Ilaria Mariani, Anna Marinello, Francesco Marrazzo, Marina Alessandra Martin, Nicolo Martinetti, Ettore Martinez, Alvise Martini, Marilena Matteo, James Mattson, Jessica Giuseppina Maugeri, Federica Mazzanti, Francesca Medici, Luca Melchior, Lorenza Menato, Beatrice Milan, Maiseloon Mogahed, Francesco Mojoli, Silvia Mongodi, Jonathan Montomoli, Giorgia Montrucchio, Lorenza Moretto, Giuseppe Moschini, Elena Munari, Giuseppe Neri, Rosella Nicoletti, Alice Nova, Nicoletta Nuzzo, Andrea Maria Olivieri, Sara Olivieri, Sebastiano Ongaro, Anita Orlando, Eman Othman, Davide Ottolina, Michele Pagani, Giacomo Paluzzano, Giulio Panciera, Valesano Paolo, Francesco Papaccio, Marcella Parente, Zoe Parimisi, Laura Pasin, Lucia Alessandra Pasqua, Federica Pavan, Matteo Perona, Paolo Persona, Tommaso Pettenuzzo, Angelo Pezzi, Elisa Pistollato, Enrico Polati, Melissa Polo Fritz, Gilda Ponzone, Matteo Pozzi, Antonella Prandini, Nicoletta Predonzani, Chiara Premoli, Davide Raimondi Cominesi, Jacopo Rama, Linda Ramahi, Ginevra Randon, Marta Repishti, Andrea Restivo, Mara Ricci, Veronica Rizzello, Erika Roat, Monica Rocco, Andrea Rodi, Egle Rondelli, Vincenzo Russotto, Debora Saggioro, Francesco Saglietti, Abdurraouf Said, Gabriele Sales, Abdulhamid Ahmed Saliga, Reem Salih, Michele Salvagno, Antonio Massimo Sammarco, Jacopo Santi, Carola Santi, Elisabetta Saraceni, Gaetano Scaramuzzo, Chiara Schiavolin, Valerio Schinetti, Vittorio Schweiger, Elena Serafini, Teodora Serana, Luca Serano, Laila Esnoussi Shalabi, Haitam Shames, Salvatore Simari, Caterina Simoni, Simone Smiraglia, Fabio Soccorsi, Alessandra Soragni, Silvana Sorrentino, Savino Spadaro, Stefano Spina, Marta Talamonti, Francesco Talarico, Chiara Natalia Tartivita, Tommaso Tenaglia, Francesco Terranova, Denise Testini, Fabio Toffoletto, Anna Toniolo, Morena Tonzar, Giulia Torsello, Tommaso Travaglini, Fabrizio Tritapepe, Luigi Tritapepe, Letizia Troisi, Fabrizio Turvani, Lucrezia Urso, Rita Vaia Liouras, Paolo Valente, Maria Sole Vallecoccia, Paola Vergano, Sara Vergine, Luigi Vetrugno, Vanessa Zambelli, Mostafa Zanaty, Francesco Zarantonello, Michela Zardin, Francesca Zini, Eugenia Zoppellaro

**Affiliations:** 1 Centre for Medical Sciences-CISMed, University of Trento, Trento, Italy; 2 UOC Anestesia e Rianimazione, AULSS8 Berica, Ospedale San Bortolo, Vicenza, Italy; 3 Institute of Anesthesia and Intensive Care, Padova University Hospital, Padua, Italy; 4 Department of Medicine and Surgery, University of Milano-Bicocca, San Gerardo Hospital, Monza, Italy; 5 Department of Precision Medicine in Medical, Surgical and Critical Care (Me.Pre.C.C.), University of Palermo, Palermo, Italy; 6 Department of Anesthesia, Intensive Care and Emergency, Policlinico Paolo Giaccone, Palermo, Italy; 7 Unit of Biostatistics, Epidemiology and Public Health, Department of Cardiac, Thoracic and Vascular Sciences, University of Padova, Padua, Italy; 8 Department of Medicine (DIMED), University of Padua, Via Vincenzo Gallucci 13, 35125 Padua, PD Italy; 9 Faculty of Medicine, University of Tripoli, Tripoli, Libya; 10 Anesthesia and Intensive Care, Ospedale All’Angelo, Mestre, Italy; 11 Anaesthesia and Intensive Care, Department of Medical and Surgical Sciences, Magna Græcia University, Catanzaro, Italy; 12 Dipartimento di Emergenza e Terapie Intensive, UOC di Anestesia e Terapia Intensiva Cardio-Toraco-Vascolare, Azienda Ospedaliera Universitaria Integrata di Verona, Verona, Italy; 13 UOC Anestesia E Rianimazione, Ospedale di Vittorio Veneto, Vittorio Veneto, TV Italy; 14 University Department of Innovative Technologies in Medicine and Dentistry, Gabriele d’Annunzio University of Chieti-Pescara, Chieti, Italy; 15 Department of Anesthesiology, Critical Care Medicine and Emergency, SS. Annunziata Hospital, Chieti, Italy; 16 Anaesthesia and Intensive Care Unit, Ospedali di San Donà di Piave e Jesolo, San Donà di Piave, Italy; 17 Department of Anesthesia and Intensive Care, Santa Maria dei Battuti-Ca’ Foncello Hospital, Treviso, Italy; 18 Anesthesia and Critical Care Unit, Ospedale di Belluno, Belluno, Italy; 19 UOC Anestesia e Rianimazione, Padova University Hospital, Padua, Italy; 20 Anesthesia and Intensive Care Unit, Manerbio Hospital, Manerbio, Italy

**Keywords:** Prone position, Mechanical ventilation, Acute respiratory failure, Arterial partial pressure of oxygen to inspired fraction of oxygen ratio (PaO_2_/FiO_2_), Ventilatory ratio, Respiratory system compliance

## Abstract

**Background:**

Prone position has been diffusely applied in mechanically ventilated COVID-19 patients. Our aim is ascertaining the association between the physiologic response and the length of the first cycle of prone position and intensive care unit (ICU) mortality.

**Methods:**

International registry including COVID-19 adult patients who underwent prone positioning. We measured the difference for arterial partial pressure of oxygen to inspired fraction of oxygen ratio (PaO2/FiO2), ventilatory ratio, and respiratory system compliance (Crs) between baseline supine position and at either the end of the first cycle of prone position (Delta-PP) or re-supination (Delta-PostPP).

**Results:**

We enrolled 1816 patients from 53 centers. Delta-PP and Delta-PostPP for PaO2/FiO2 were both associated with ICU mortality [OR (95% CI) 0.48 (0.38, 0.59), and OR (95% CI) 0.60 (0.52, 0.68), respectively]. Ventilatory ratio had a non-linear relationship with ICU mortality for Delta-PP (p = 0.022) and Delta-PostPP (p = 0.004). Delta-PP, while not Delta-PostPP, for Crs was associated with ICU mortality [OR (95% CI) 0.80 (0.65, 0.98)]. The length of the first cycle of prone position showed an inverse relationship with ICU mortality [OR (95% CI) 0.82 (0.73, 0.91)]. At the multivariable analysis, the duration of the first cycle of prone position, Delta-PP and Delta-PostPP for PaO2/FiO2, and Delta-PostPP for ventilatory ratio were independently associated with ICU mortality.

**Conclusion:**

In COVID-19 patients with acute respiratory failure receiving invasive mechanical ventilation and prone positioning, the physiological response to prone position is associated with ICU mortality. Prolonging the duration of the first cycle of prone position is associated with improved survival.

**Supplementary Information:**

The online version contains supplementary material available at 10.1186/s13613-025-01438-y.

## Background

In patients with moderate-to-severe acute respiratory distress syndrome (ARDS) receiving invasive mechanical ventilation, prone position may improve arterial blood gases through different mechanisms such as rearrangement of the gas–tissue ratios along the dependent–nondependent regions of the lungs, enhanced ventilation/perfusion matching, and a more homogeneous distribution of lung stress and strain [1]. A large multicenter randomized trial found that prone positioning within 24 h after the diagnosis of ARDS and for at least 16 h for the first maneuver and 12 h for the following ones reduced both 28-day and 90-day mortality in moderate-to-severe ARDS patients [2]. Afterwards, other studies and meta-analyses confirmed a survival benefit of prone positioning in moderate-to-severe ARDS [3–5], so that since 2017 prone position has been strongly recommended with a high level of evidence for patients with severe or moderate-to-severe ARDS to reduce mortality [6–8].

Nonetheless, before the coronavirus disease 2019 (COVID-19) pandemic, prone position struggled to become part of the routine clinical practice for the management of patients with moderate-severe ARDS, where prone positioning was implemented in less than 1 out of 10 cases within 48 h [9] and not even performed in almost 70% of patients with severe ARDS prior to veno-venous extracorporeal membrane oxygenation (V-V ECMO) cannulation in experienced centers [10].

Quite the opposite, prone positioning has been diffusely adopted during the COVID-19 pandemic. In fact, prone position was used in 76% of 735 COVID-19-related ARDS patients included in a multicenter cohort in Spain [11], and in 61% of 1057 patients on invasive mechanical ventilation for COVID-19 acute respiratory failure in an Italian multicenter cohort study [12].

During the COVID-19 pandemic we set up an international multicenter registry, the Prone Positioning for Invasively Ventilated Patients with COVID-19 (PROVENT-C19) Registry [13], which aimed at ascertaining the association between Intensive Care Unit (ICU) mortality and the response to the first cycle of prone position, as assessed by physiological parameters [1] such as arterial oxygenation, dead space fraction estimates, and respiratory system compliance. Secondly, we aimed at profiling patients based on the physiological response to prone position in a large cohort of patients experiencing COVID-19 acute respiratory failure. Moreover, we investigated the associations between ICU mortality and both the length of the first cycle of prone position and the total number of prone position cycles.

## Methods

The PROVENT-C19 is a multicenter, observational registry developed by the Veneto ICU Network [14], and endorsed by the Italian Society of Anesthesia, Analgesia, Resuscitation and Intensive Care, the European Society of Intensive Care Medicine, and the European Society of Anaesthesiology and Intensive Care [13]. There is no funding source.

The registry was designed following the Declaration of Helsinki and the study protocol was firstly approved by the Vicenza Ethics Committee (ID:22/21). Informed consent was obtained according to the European Union General Data Protection Regulation, the national regulations or local directives of each participating Institution. In cases the patient was incompetent because of critical illness or the use of sedative or anesthetic drugs, consent could be delayed, and a provision for delayed consent was applied: as soon as competent, each patient was fully informed on what had been done, and a written permission of using data collected was obtained. The patients or their legal surrogates were informed of their right to request that the study procedures be discontinued and their right to refuse the study-related use of their medical records.

The Strengthening the Reporting of Observational studies in Epidemiology reporting guideline checklist for observational studies was used for reporting this study (Supplementary Material 1).

The registry included, either prospectively or retrospectively, consecutive adult patients who underwent invasive mechanical ventilation and prone positioning due to COVID-19 related acute respiratory failure, from December 31st 2019 to January 1st 2023. Patients were excluded if they refused the consent to participate or if they presented contraindications to prone position [1, 15].

**Fig. 1:**
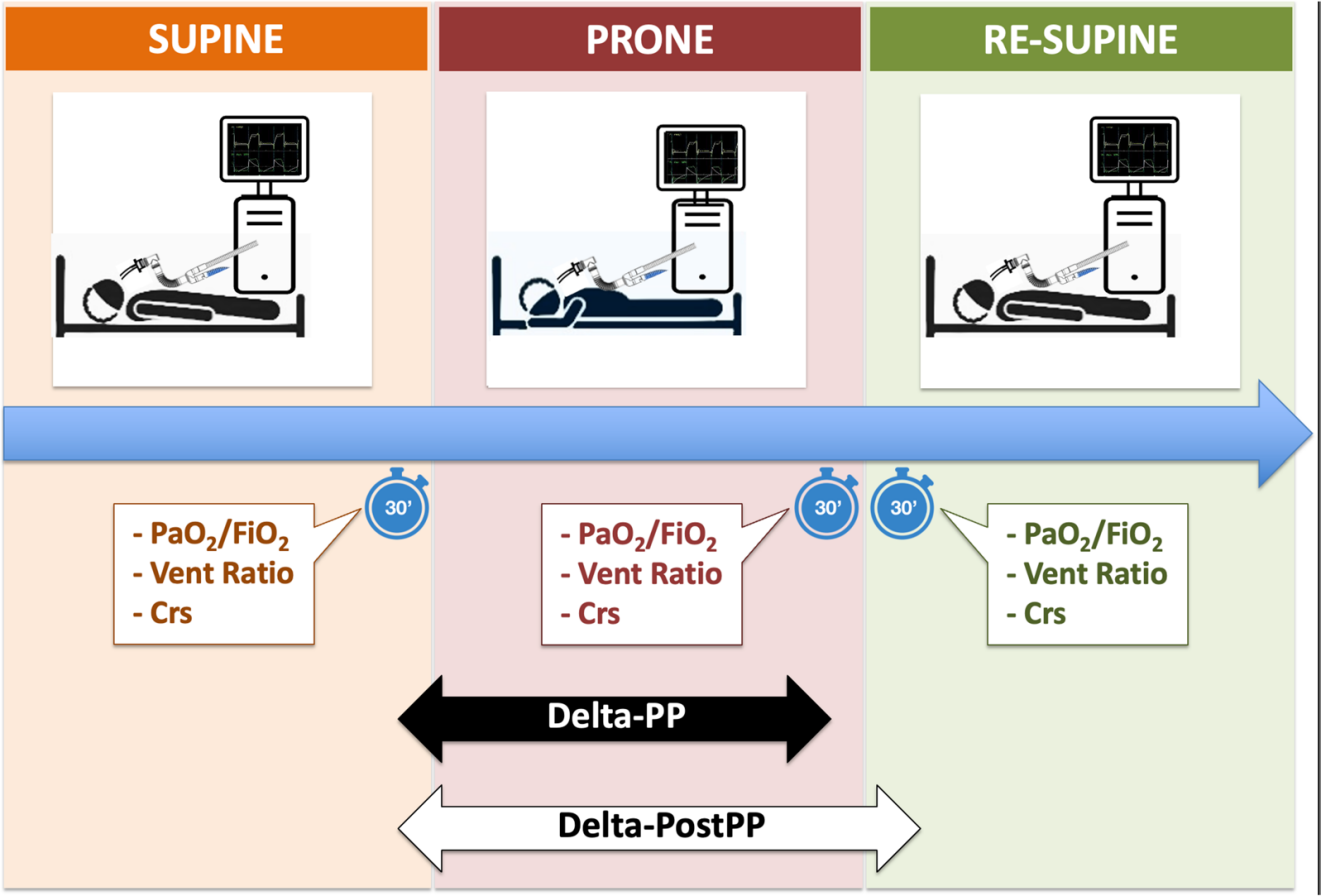
Study design and timeline. These time windows were desirable but not obligatory. The real timing of variable collection was recorded and variables registered more than 30 min later than required by the study design were excluded. PaO_2_/FiO_2_ arterial partial pressure of oxygen to inspire fraction of oxygen ratio. Vent Ratio, ventilatory ratio. Crs, static compliance of the respiratory system

Physiological response to prone position was evaluated at different time points (Fig. 1), calculating for arterial partial pressure of oxygen to fraction of inspired oxygen ratio (PaO_2_/FiO_2_), ventilatory ratio (as proxy for dead space) [16, 17], and static compliance of the respiratory system [Crs]) [18–20]:

- Difference in prone position (Delta-PP): the difference between the last available values within the last 30 min in prone and supine position (prior to being turned prone), respectively;
- Difference after prone positioning (Delta-PostPP): the difference between the first available value within the first 30 min in supine position after the end of the prone position cycle and the last available value in supine position before being turned prone (in the last 30 min prior to being turned prone).

Patients were categorized according to:

- ICU survival;
- length of the first cycle of prone position in three groups: (1) short pronation (i.e., < 16 h); (2) long pronation (between 16 and 24 h); and (3) extended pronation (i.e., > 24 h).

### Statistical analysis

Complete case analysis was performed.

Categorical data are presented as absolute numbers (n) and percentages (%). For continuous data medians and I and III quartiles are used. The distribution of categorical variables was compared with the Pearson's Chi-squared test or the Fisher's exact test, whichever appropriate, while the distribution of continuous variables was compared using Wilcoxon rank sum test. To account for multiplicity of testing, Benjamini–Hochberg correction was employed.

Univariable logistic regression models were employed to assess the association between patients' characteristics or physiological response to prone position and ICU mortality. For nonlinear association, restricted cubic splines were used. To account for clustering effect within the same center, the Huber-White method was employed.

A multivariable logistic regression model was used to evaluate the association between physiological response to prone position and ICU mortality after adjusting for relevant confounders, including the ICU admission period.

A subgroup analysis was conducted to assess the consistency of findings in retrospective and prospective data: logistic regression models for ICU mortality were fitted, including interaction terms between Delta-PP and recruitment type (prospective vs. retrospective) and between Delta-PostPP and recruitment type (prospective vs. retrospective).

All statistical tests were 2-tailed, and statistical significance was defined as *p* < 0.05. All analyses have been conducted using R 4.0.3 (Vienna, Austria).

Additional details on methods and statistics are provided in the Supplementary Material 2.

## Results

A total of 1816 patients were enrolled from 53 international centers (Supplementary Material 3 and Supplementary Material 4). The baseline characteristics of the study population are shown in Table 1 and Supplementary Material 5, while the main clinical outcomes are listed in Table 2. Overall, 837 patients (47.7%) were discharged alive from ICU, while 916 patients (52.3%) died during the ICU stay. The risk factors associated with ICU mortality are reported in Tables 1 and 2.

**Table 1:** Demographic and baseline characteristics of the study population

Characteristics	Overall	ICU survivors	ICU non-survivors	OR (95% CI)	P-value
	N	n = 837 (47.7%)	n = 916 (52.3%)		
*Demographics*					
Age, *years*	1752	61(53, 69)	69 (62, 74)	2.47 (1.92, 3.18)	** < 0.001**
Male, *n (%)*	1750	576 (69.0)	659 (72.0)	1.16 (0.98, 1.36)	0.077
BMI, *kg/m*^*2*^	1667	29 (26, 33)	28 (25, 31)	0.94 (0.84, 1.05)	0.269
Pandemic wave, *n (%)*:	1753				
- 1st wave (January 2020–August 2020)		94 (11.0)	70 (7.6)	–	–
- 2nd wave (September 2020–March 2021)		362 (43.0)	462 (50.0)	1.71 (1.22, 2.41)	**0.002**
- 3rd wave (April 2021–December 2022)		381 (46.0)	384 (42.0)	1.35 (0.96, 1.91)	0.081
*Comorbidities*					
COPD, *n(%)*	1751	41 (4.9)	127 (14.0)	3.12 (2.01, 4.84)	** < 0.001**
Arterial hypertension, *n (%)*	1752	393 (47.0)	580 (63.0)	1.94 (1.54, 2.44)	** < 0.001**
Chronic heart failure, *n (%)*	1752	69 (8.3)	164 (18.0)	2.42 (1.69, 3.46)	** < 0.001**
Cerebral vasculopathy, *n (%)*	1752	23 (2.8)	48 (5.2)	1.95 (1.12, 3.42)	**0.019**
Diabetes mellitus, *n (%)*	1752	172 (21.0)	270 (29.0)	1.61 (1.26, 2.06)	** < 0.001**
Chronic kidney disease, *n (%)*	1751	11 (1.3)	67 (7.3)	5.93 (2.78, 12.62)	** < 0.001**
Home renal replacement therapy, *n (%)*	1751	5 (0.6)	11 (1.2)	0.31 (0.08, 1.24)	0.097
Chronic liver failure, *n (%)*	1752	9 (1.1)	24 (2.6)	2.47 (1.18, 5.17)	**0.016**
Cancer, *n (%)*	1752	26 (3.1)	53 (5.8)	1.91 (1.11, 3.30)	**0.020**
Immunological deficiency, *n (%)*	1751	41 (4.9)	91 (9.9)	2.14 (1.43, 3.20)	** < 0.001**
*Before ICU admission*					
COVID-19 vaccination, *n (%)*	1232	83 (14.0)	99 (15.0)	1.07 (0.71, 1.60)	0.750
Hospitalization before ICU admission, *days*	1752	2 (1, 5)	3 (1, 7)	1.00 (0.99, 1.00)	0.372
Corticosteroids before ICU admission, *n(%)*	1745	566 (68.0)	655 (72.0)	1.22 (0.80, 1.86)	0.354
Anticoagulant therapy before ICU admission, *n (%)*	1744	542 (65.0)	610 (67.0)	1.10 (0.76, 1.61)	0.611
Non-invasive respiratory support before IMV, *n (%)*	1749	728 (87.0)	790 (87.0)	0.97 (0.65, 1.46)	0.881
Non-invasive respiratory support before IMV, *days*	1326	2 (1, 4)	4 (2, 7)	1.57 (1.28, 1.93)	** < 0.001**
*ICU admission*					
IMV at ICU admission, *n (%)*	1751	213 (25.0)	306 (33.0)	1.41 (0.85, 2.35)	0.184
PaO_2_/FiO_2_ at ICU admission, *mmHg*	1742	90 (69, 118)	84 (65, 114)	0.92 (0.82, 1.02)	0.108
Glasgow Coma Scale at ICU admission	1711	15 (15, 15)	15 (14, 15)	0.45 (0.13, 1.57)	0.212
SOFA at ICU admission	1720	4 (3, 5)	4 (4, 7)	1.54 (1.25, 1.90)	** < 0.001**
White Blood Cells at ICU admission, × *10*^*9*^*/L*	1735	10 (7, 13)	11 (8, 15)	1.00 (0.99, 1.01)	0.315
CRP at ICU admission, *mg/L*	1413	48 (13, 130)	38 (11, 125)	0.97 (0.76, 1.25)	0.839
Procalcitonin at ICU admission, *mcg/L*	1244	0.20 (0.10, 0.50)	0.26 (0.12, 0.78)	1.01 (1.00, 1.02)	0.189
D-Dimer at ICU admission, *mcg/L*	1178	912 (354, 2020)	1099 (35, 3898)	1.03 (0.99, 1.07)	0.124

Results of the univariable analysis

Data are median (I quartile–III quartile) for continuous variables and absolute numbers (percentages) for categorical variables. Results of the univariable analyses are reported as Odds Ratio (OR), 95% Confidence Interval (CI), *p-value*

*ICU* intensive care unit, *OR* Odds Ratio, *95%CI* 95% Confidence Interval, *BMI* body mass index, *COPD* chronic obstructive pulmonary disease, *IMV* invasive mechanical ventilation, *PaO*_*2*_*/FiO*_*2*_ arterial partial pressure of oxygen to inspire fraction of oxygen ratio, *SOFA* sequential organ failure assessment, *CRP* C-reactive protein

**Table 2:** Main clinical outcomes of the study population

Characteristics	Overall	ICU survivors	ICU non-survivors	OR (95% CI)	P-value
	N	n = 837 (47.7%)	n = 916 (52.3%)		
*Outcomes*					
ICU LOS, *days*	1748	16 (10, 28)	13 (8, 22)	0.90 (0.79, 1.02)	0.091
Hospital LOS, *days*	1713	35 (22, 52)	19 (12, 28)	0.45 (0.30, 0.67)	** < 0.001**
28-day ventilator free days	992	15 (3, 20)	0 (0, 0)	0.58 (0.50, 0.64)	** < 0.001**
Tracheostomy, *n (%)*	1751	265 (32.0)	197 (22.0)	0.59 (0.40, 0.87)	**0.007**
Renal replacement therapy, *n (%)*	1741	33 (4.0)	151 (17.0)	4.81 (2.89, 8.01)	** < 0.001**
ECMO or ECCO_2_R, *n (%)*	1727	19 (2.3)	27 (3.0)	1.30 (0.54, 3.14)	0.565
iNO, *n (%)*	1631	49 (6.2)	52 (6.2)	1.00 (0.56, 1.79)	0.998
Cycles of prone position, *n (%)*	1753	2 (2, 4)	3 (2, 5)	1.06 (0.92, 1.11)	0.102
Overall time in prone position, *hours*	1651	64 (40, 98)	63 (38, 102)	1.02 (0.85, 1.23)	0.835
Hospital mortality,* n (%)*	1720	39 (4.8)	\	\	\

Results of the univariable analysis

Data are median (I quartile-III quartile) for continuous variables and absolute numbers (percentages) for categorical variables. Results of the univariable analyses are reported as Odds Ratio (OR), 95% Confidence Interval (CI), *p-value*

*ICU* intensive care unit, *OR* Odds Ratio, *95%CI* 95% Confidence Interval, *LOS* length of stay, *ECMO* extracorporeal membrane oxygenation, *ECCO*_*2*_*R* extracorporeal carbon dioxide removal, *iNO* inhaled nitric oxide

### Physiological response to prone position

**Fig. 2:**
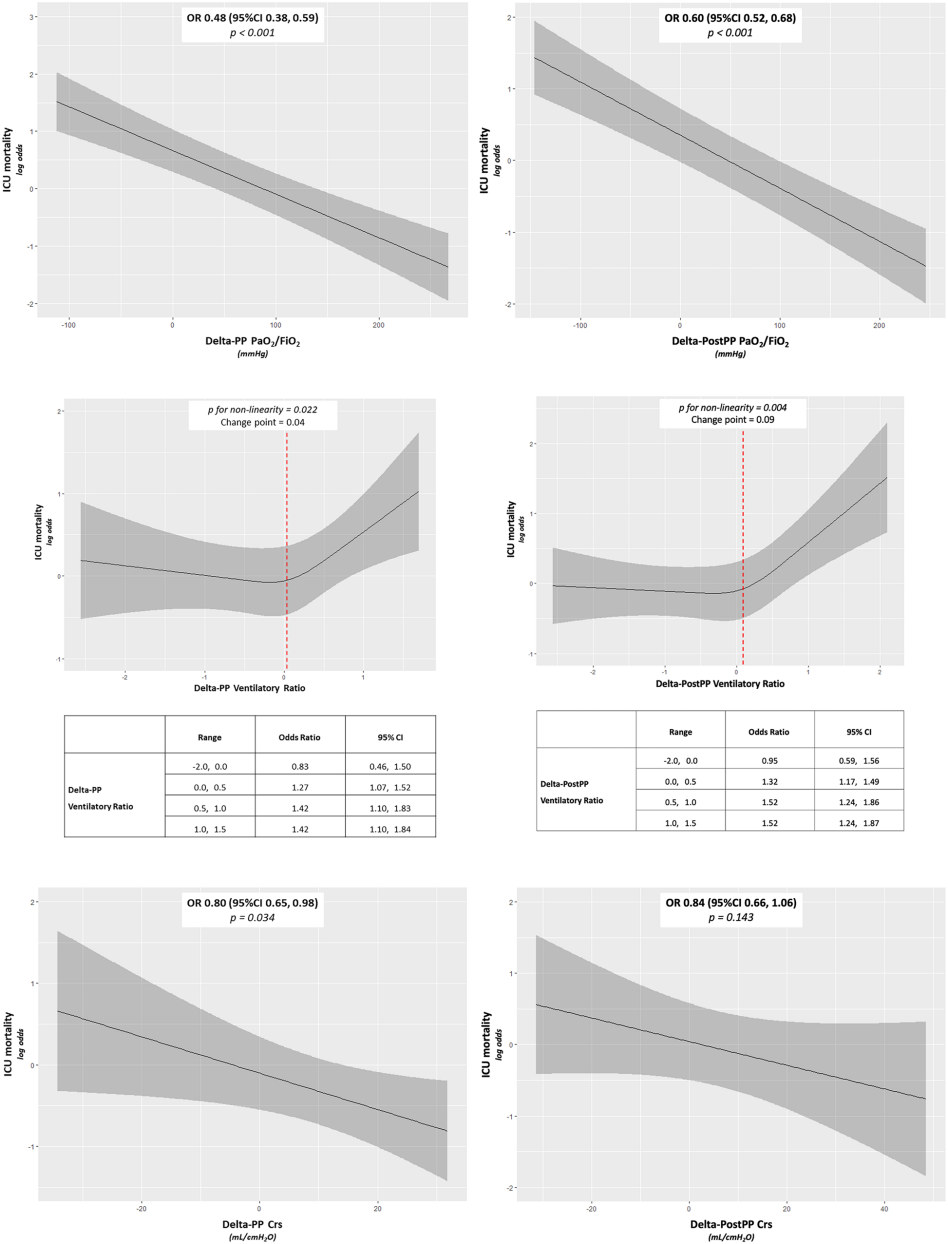
Relationship between ICU mortality and the response to the first cycle of prone position on the basis of arterial oxygenation at the end of the prone position cycle (Delta-PP) (**A**) and after re-supination (Delta-PostPP) (**B**), on the basis of ventilatory ratio at the end of the prone position cycle (Delta-PP) (**C**) and after re-supination (Delta-PostPP) (**D**), and on the basis of respiratory system static compliance at the end of the prone position cycle (Delta-PP) (**E**) and after re-supination (Delta-PostPP) (**F**). The x-axis shows the predictor and the y-axis shows the effect of the predictor on the outcome in log-odds. The solid line represents the regression line estimated by the logistic regression model, and the gray bands show the confidence intervals. When the relationship was found to be non-linear, the change-point was identified (red dotted line). Log-odds quantify the influence of factors on the probability of an outcome: a positive value indicates that the probability of the event increases with the factor, while a negative value suggests a decrease. If we suppose the logistic regression model gives a log-odds for Delta-PP for PaO_2_/FiO_2_ of −0.05, this log-odds value indicates that for each additional point of Delta-PP for PaO_2_/FiO_2_, the log-odds of death in the ICU decreases by 0.05. In panel **A**, the odds ratio of 0.48 for ICU mortality associated with an increase of Delta-PP for PaO_2_/FiO_2_ indicates that an improvement in the PaO_2_/FiO_2_ ratio within the interquartile range (i.e., from 26 to 124 mmHg) significantly reduces the odds of ICU mortality. Specifically, this increase from the 25th to the 75th percentile is associated with a 52% reduction in the odds of ICU mortality. The same rule could be applied for the interpretation of the linear relationships depicted in panels **B**, **E**, and **F.** When the relationship is non-linear, as in panels** C** and** D**, the odds ratio for ICU mortality refers to an increase of the variable within the range indicated in the table below the chart. For example, in panel **C**, the odds ratio of 1.27 for ICU mortality associated with the increase of Delta-PP for ventilatory ratio from 0.0 to 0.5 indicates that the odds of ICU mortality are significantly increased if Delta-PP for ventilatory ratio passes from 0.0 to 0.5. PaO_2_/FiO_2_ arterial partial pressure of oxygen to inspire fraction of oxygen ratio. Crs, static compliance of the respiratory system. OR, odds ratio. 95%CI, 95% confidence interval

The response to the first cycle of prone position on the basis of PaO_2_/FiO_2_ was available for 1655 patients. As depicted in Fig. 2A, B, both Delta-PP and Delta-PostPP for PaO_2_/FiO_2_ showed an inverse linear relationship with ICU mortality [OR (95% CI) 0.48 (0.38, 0.59), p < 0.001, and OR (95% CI) 0.60 (0.52, 0.68), p < 0.001, respectively]. By plotting Delta-PostPP for PaO_2_/FiO_2_ versus Delta-PP for PaO_2_/FiO_2_, patients were divided in four quadrants according to their oxygenation responses (improvement or worsening) to the first cycle of prone position (Supplementary Material 6). The group who experienced an increase in PaO_2_/FiO_2_ both at the end of prone position (Delta-PP > 0 mmHg) and at re-supination (Delta-PostPP > 0 mmHg) presented the lower ICU mortality [47.0 (44.0—50.0) %]. Worth remarking, patients underwent further cycles of prone positioning in the vast majority of cases, either in case Delta-PP for PaO_2_/FiO_2_ improved (92%) or not (87%) within the first cycle of prone position.

Secondly, the response to the first cycle of prone position with respect to the ventilatory ratio was assessed in 1509 patients. As depicted in Fig. 2C, D, both Delta-PP and Delta-PostPP for the ventilatory ratio showed a non-linear relationship with ICU mortality (p-value for non-linearity = 0.022 and 0.004, respectively), with a linear increase of the risk of death in ICU at increasing values of Delta-PP and Delta-PostPP for ventilatory ratio only for values greater than 0.04 and 0.09, respectively.

Finally, the response to the first cycle of prone position on the basis of Crs was available in 469 patients. As depicted in Fig. 2E, Delta-PP for Crs showed an inverse linear relationship with ICU mortality [OR (95% CI) 0.80 (0.65, 0.98), p = 0.034]. Delta-PostPP for Crs exhibited a trend towards an inverse relationship with ICU mortality, but the association was not significant [OR (95% CI) 0.84 (0.66, 1.06), p = 0.067] (Fig. 2F).

### Duration and frequency of prone position cycles

**Fig. 3:**
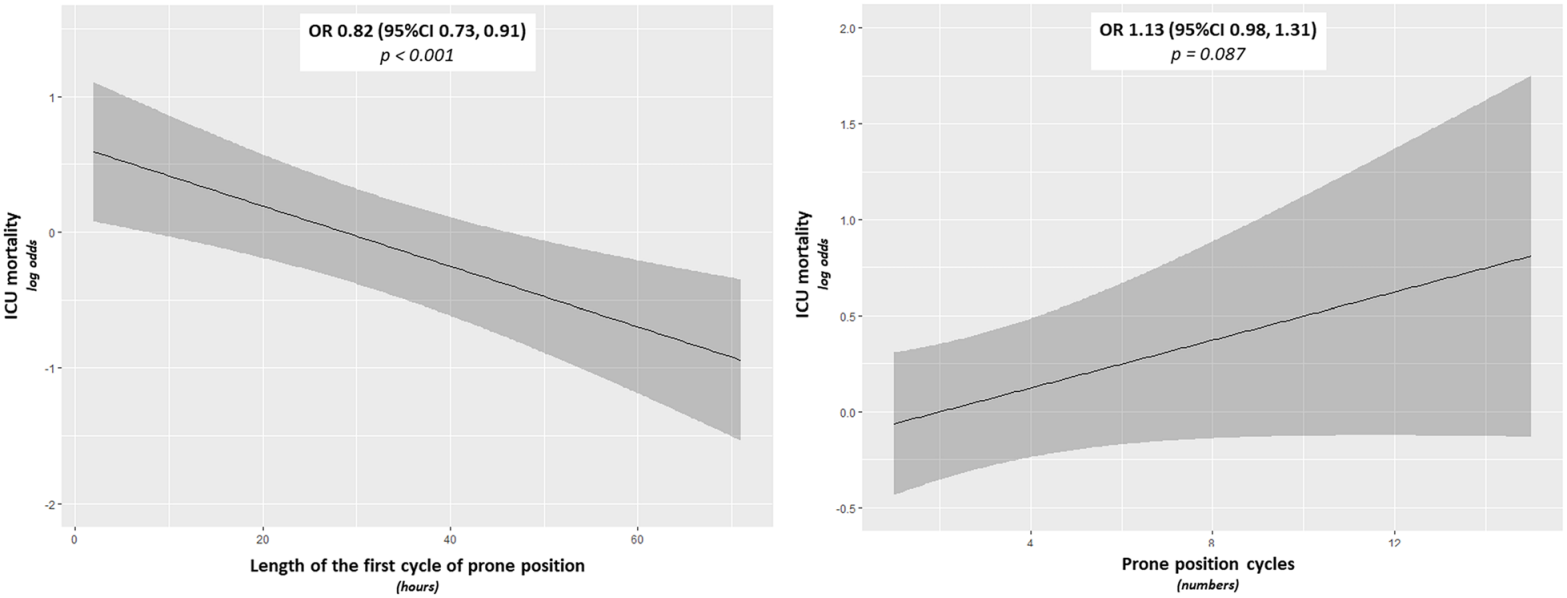
Relationship between ICU mortality and the length of the first cycle of prone position (**A**) and the total numbers of prone position cycles (**B**). The x-axis shows the predictor and the y-axis shows the effect of the predictor on the outcome in log-odds. The solid line represents the regression line estimated by the logistic regression model, and the gray bands show the confidence intervals. Log-odds quantify the influence of factors on the probability of an outcome: a positive value indicates that the probability of the event increases with the factor, while a negative value suggests a decrease. If we suppose the logistic regression model gives a log-odds for the length of prone position of −0.05, this log-odds value indicates that for each additional point of prone position duration, the log-odds of death in the ICU decreases by 0.05. OR, odds ratio. 95%CI, 95% confidence interval

The length of the first cycle of prone position showed a linear inverse relationship with ICU mortality [OR (95% CI) 0.82 (0.73, 0.91), p = 0.001], while the frequency of the prone position cycles was not associated with ICU mortality [OR (95% CI) 1.13 (0.98, 1.31), p = 0.087] (Fig. 3). ICU mortality did not differ significantly between those who were not treated with further cycles of prone positioning after the first one and those who underwent multiple cycles (46% vs 53%, respectively, p = 0.186).

The baseline and clinical characteristics and the main outcomes of patients according to the short, long or extended pronation group are shown in Supplementary Material 7. Worth remarking, short pronation was more frequently adopted during the first wave of COVID-19 pandemic (January 2020–August 2020), accounting for 44.1% of the overall patients registered in this period (p < 0.001), while extended pronation was more extensively applied during the third wave (April 2021–December 2023), accounting for 17.4% of the overall patients recorded in this period (p < 0.001). The short pronation group experienced significantly lower Delta-PP for PaO_2_/FiO_2_ [(45 (6–95) mmHg] compared to the long [73 (33–125) mmHg] and extended pronation group [72 (30, 125) mmHg] (p < 0.001). Likewise, the extended pronation group showed the greatest Delta-PostPP for PaO_2_/FiO_2_ [39 (11–85) mmHg] compared to the other two groups [17 (− 1–58) mmHg, and 28 (− 1–66) mmHg, respectively] (p < 0.001). Conversely, no differences between the three groups were identified in Delta-PP and Delta-PostPP for the ventilatory ratio and Crs.

As depicted in Supplementary Material 8, a non-linear relationship was found between the length of the first cycle in prone position and the oxygenation response as assessed by Delta-PP for PaO_2_/FiO_2_ (p-value for non-linearity < 0.001), with a linear increase of Delta-PP at increasing duration up to 19 h. The length of the first cycle of prone position and Delta-PostPP for PaO_2_/FiO_2_ showed a direct linear relationship [OR (95% CI) 7.21 (3.20, 11.23), p < 0.001].

### Multivariable analysis for ICU mortality

At the multivariable analysis, in the model 1 both the duration of the first cycle of prone position [OR (95% CI) 0.84 (0.72, 0.96)] and Delta-PP for PaO_2_/FiO_2_ [OR (95% CI) 0.51 (0.41, 0.63)] were confirmed to be independently associated with ICU mortality, while not Delta-PP for ventilatory ratio [OR (95% CI) 1.07 (0.95, 1.20)]. In the model 2 the duration of the first cycle of prone position [OR (95% CI) 0.82 (0.71, 0.95)], Delta-PostPP for PaO_2_/FiO_2_ [OR (95% CI) 0.58 (0.47, 0.71)], and Delta-PostPP for ventilatory ratio [OR (95% CI) 1.20 (1.05, 1.37)] were all independently associated with ICU mortality (Table 3).

**Table 3:** Results of the multivariable analysis for ICU mortality

n = 945	Variable interquartile range	ODDS ratio	95% CI
*MODEL 1*			
Age, *years*	57, 73		
Arterial hypertension (Ref. No)	\		
Chronic heart failure (Ref. No)	\		
Pandemic wave	\		
SOFA at ICU admission (unit increase)	3, 6		
D-Dimer at ICU admission, *mcg/L*	285, 2737		
Length of the 1st cycle in prone position, *hours*	16, 25	**0.84**	**0.72, 0.96**
Delta-PP for PaO_2_/FiO_2_, *mmHg*	26, 124	**0.51**	**0.41, 0.63**
Delta-PP for ventilatory ratio	− 0.15, 0.28	1.07	0.95, 1.20
*MODEL 2*			
Age, *years*	57, 73		
Arterial hypertension (Ref. No)	\		
Chronic heart failure (Ref. No)	\		
Pandemic wave	\		
SOFA at ICU admission (unit increase)	3, 6		
D-Dimer at ICU admission, *mcg/L*	285, 2737		
Length of the 1st cycle in prone position, *hours*	16, 25	**0.82**	**0.71, 0.95**
Delta-PostPP for PaO_2_/FiO_2_, *mmHg*	1, 70	**0.58**	**0.47, 0.71**
Delta-PostPP for ventilatory ratio	− 0.15, 0.38	**1.20**	**1.05, 1.37**

Data are Odds Ratio, 95% Confidence Interval (CI)

*95%CI* 95% Confidence Interval, *SOFA* sequential organ failure assessment, *ICU* intensive care unit, *PaO*_*2*_*/FiO*_*2*_ arterial partial pressure of oxygen to inspire fraction of oxygen ratio

### Subgroup analysis according to recruitment type

As reported in Supplementary Material 9, the effects of Delta-PP and Delta-PostPP on ICU mortality did not differ between the retrospective and prospective subgroups.

## Discussion

In this multicenter international registry, exploring a large cohort of mechanically ventilated COVID-19 patients with acute respiratory failure, we found the physiological response to prone position to be associated with ICU mortality. The oxygenation improvement, observed either at the end of the first cycle of prone position or after re-supination, was inversely associated with ICU mortality, with the lowest death rate in patients experiencing oxygenation improvement both during prone position and after re-supination. Moreover, we showed that the worsening of either dead space or respiratory system compliance during the first cycle of prone position was associated with a higher risk of ICU mortality. Finally, in our study population the longer the duration of the first prone position cycle, the smaller the ICU mortality. After adjustment for clinically meaningful variables, both an improvement in oxygenation and the duration of the first prone positioning confirmed to be independently associated with ICU mortality.

In COVID-19 patients, Camporota and colleagues reported that the increase in oxygenation upon prone position was an independent predictor of ICU survival [21]. Imputed mechanisms of improved oxygenation during prone position include a higher ratio between the resolution of dorsal atelectasis and the onset of ventral atelectasis—as described by computed tomography data [22]—and improved ventilation perfusion matching [15, 23]. In our population we confirmed the positive effects of prone position on oxygenation and its independent association with ICU mortality. These findings differ from the secondary analysis of the Proseva trial by Albert et al. [24], who did not observe any association between the improvement in arterial oxygenation during prone positioning and survival in 232 patients randomized to receive prone position for severe ARDS. Differences in sample size, etiology of respiratory failure, and timing of oxygenation assessment might be factors explaining this discrepancy. Although our results show an association between improved oxygenation at End-PP and mortality, we cannot infer causality, and we do not suggest that clinical decision making be based on oxygenation response.

Furthermore, we observed a significant effect of prone position in improving oxygenation—as compared to baseline supine position—after re-supination. Of note, the improved oxygenation upon prone position showed the lowest mortality when associated with a persistent improvement in oxygenation after re-supination.

This effect was probably obtained through an improvement in respiratory mechanics during prone position, such as an increased end expiratory lung volume and a decreased strain, as reported by Dilken et al. [25]. In keeping with these findings, we observed that an improved Crs linearly predicted a decrease in ICU mortality. This is in line with what has already been observed in severe ARDS patients, who showed improved survival when prone position improved Crs [26]. The main mechanism advocated to explain the improvement of Crs in prone position is the recruitment of the dorsal region of the lung during prone position [1].

In keeping with Gattinoni et al. who showed the response on carbon dioxide clearance after prone positioning to be directly correlated with survival in non-COVID-19 ARDS patients [27], we found ICU mortality to be linearly associated with a decreasing efficiency of alveolar ventilation and we observed a strong signal towards an increased ICU mortality in patients with greater ventilatory ratio after the first cycle of prone position, for both Delta-PP and Delta-PostPP. For instance, ventilatory ratio is a readily accessible bedside respiratory variable that functions as a proxy for physiologic dead space. Like dead space, ventilatory ratio also is correlated with mortality in ARDS, being an elevated ventilatory ratio associated with poorer outcomes in patients with ARDS [16, 28]. Recently, da Cruz et al. described the presence of 2 different COVID-19 phenotypes based on longitudinal respiratory variables and the one associated with a higher mortality rate was characterized by a higher ventilatory ratio during the first prone session [29]. Our findings, although associative, corroborate the potential beneficial implications of assessing the response in ventilatory ratio during and after prone positioning on outcomes. The increase in physiologic dead space as a response to prone positioning—as indicated by a higher ventilatory ratio—may introduce the concept of negative responders to prone positioning [27], which remains to be confirmed by future dedicated trials with causative design. If so, this new insight in the physiological response to prone position may have two clinical implications: (1) the increase in ventilatory ratio consequent to prone positioning may be utilized as a prognostic variable to infer an increase in mortality; (2) continuation of prone positioning should be carefully evaluated after a response in increased ventilatory ratio. This may serve as a future area of investigation to explore whether the increase of dead space secondary to lung recruitment maneuvers (e.g., prone positioning) may guide lung recruitment or explain an increased mortality in respiratory failure.

Since the cornerstone study by Guerin et al., which led to the guidelines recommendation on appling one 16-h session followed by 12-h sessions of prone position in ARDS patients [2], the COVID-19 pandemic introduced the implementation of prolonged/extended duration of prone position, which resulted to be relatively safe and potentially more effective [1, 30–32].

Despite a trend toward a higher rate of pressure ulcers in the extended prone position subgroup (≥ 24 h) as compared with subgroups receiving shorter durations, not achieving, nonetheless, statistical significance, our data overall confirm that prolonged/extended prone position does not add harm. We found that prolonged prone position significantly improved oxygenation both for Delta-PP and Delta-PostPP, indicating an exposure-dependent response to prone position in this cohort of patients [33]. Noteworthy, while the duration of the first cycle of prone positioning was linearly associated to a higher response in oxygenation during prone position up to a plateau of 19 h, it was linearly associated with a constantly increasing oxygenation after re-supination.

More importantly, our data demonstrated for the first time in a large population an inverse linear relationship between length of application of the first prone position and ICU mortality, in keeping with the findings by Okin et al. who showed in mechanically ventilated COVID-19 hypoxemic patients that cycles ≥ 24 h reduced short- and long-term mortality [30].

This international multicenter clinical registry has strengths, including (1) the large sample size; (2) the high numbers of centers involved in the study which helps a high generalizability of the study findings; and (3) the noticeable data granularity.

We would be remiss not to mention some of the limitations of our study. First, it is a real-life registry that bears all the limitations of this study design. In particular, the study did not comprehensively explore potential confounding factors and sources of bias, such as variations in patient management protocols (e.g., implementation of awake prone positioning) and inconsistencies in data recording practices across different healthcare facilities. However, to limit the cohort effect due to the dynamics of disease characteristics and clinical management of critically ill COVID-19 patients over the time course of the pandemic [34], the multivariable analysis was corrected to account for the potential confounding effect of the ICU admission period. Second, since the registry included either prospective or retrospective data, information bias related to the retrospective nature of some of the collected data cannot be ruled out. Third, since our study focused on COVID-19 patients only, whether or not these results may be extended to other patient populations remains to be ascertained. However, allowing for the first time the collection of large amounts of data in an homogenous population of patients with acute hypoxemic respiratory failure caused by the same disease, COVID-19 represented a good model for studying acute respiratory failure secondary to viral pneumonia, despite some specific characteristics of the COVID-19. Furthermore, differences in hospital resources and surge during the COVID-19 pandemic among different hospitals cannot be excluded and this may have been linked to patients' outcomes. Indeed, variations in the duration of the first cycle may be driven by variations in "quality" and capacity of care in each ICU: if an ICU is short-staffed, overworked, or facing a surge of patients, personnel may be not be able to prone for long periods, thus patients could be more likely to die just because staff is overwhelmed and less attentive to react to complications. Whether or not this may have played a role in the number and duration of prone position cycles during the daily care and their potential impact on ICU mortality cannot be ascertained. Last, considering the design of the study, mechanisms underlying the physiological and clinical response to prone positioning are not investigated. These limitations underscore the need for cautious interpretation of the findings and highlight areas for further research to enhance our understanding of the topic.

### Implication for practice

The findings of this large multicenter study may have implications for clinical practice. Indeed, our results seem to suggest that either an improvement of arterial oxygenation at the end of the first cycle of prone position, especially when maintained after re-supination, or an increase of Crs in prone position seem to be favorable prognostic indexes for hypoxemic patients with acute respiratory failure receiving invasive mechanical ventilation and prone positioning.

Furthermore, because a longer duration of the first prone position cycle is associated with a greater improvement in arterial oxygenation after re-supination and a lower ICU mortality without major adverse effects, it may be reasonable to maintain a patient as long as possible in prone position in order to maximize its beneficial effects and reduce ICU mortality, and it is justified to address the lengthening of PP in large scale randomized clinical trials.

## Conclusions

In this large international multicenter clinical registry including COVID-19 patients with acute respiratory failure receiving invasive mechanical ventilation and undergoing prone positioning, we found the physiological response to prone position to be associated with ICU mortality. Furthermore, prolonging the duration of the first cycle of prone position seemed to improve oxygenation and survival. Whether or not these results may be extended to other patient populations remains to be ascertained.

## Supplementary Information

Supplementary Material 1. The Strengthening the Reporting of Observational studies in Epidemiologyreporting guideline checklist

Supplementary Material 2. Extended Methods

Supplementary Material 3. Patients enrollment among the participating centers

Supplementary Material 4. Study patients flowchart. Abbreviations. ICU, intensive care unit. PaO_2_/FiO_2_ arterial partial pressure of oxygen to inspire fraction of oxygen ratio. *Combined enrollment indicates patients who were included in the registry after the end of the first cycle of prone position, but before hospital discharge, thus part of the data has been collected retrospectively, while other information has been registered prospectively.

Supplementary Material 5. Main clinical characteristics of the study population. Data are median for continuous variables and absolute numbers for categorical variables

Supplementary Material 6. Relationship between the arterial oxygenation response to the first cycle of prone positioning evaluated at the end of prone position before and after being turned supine. Abbreviations. PaO_2_/FiO_2_ arterial partial pressure of oxygen to inspire fraction of oxygen ratio. 95%CI, 95% confidence interval. ICU, intensive care unit.

Supplementary Material 7. Baseline characteristics and main clinical outcomes of the study population stratified according to the length of the first cycle in prone position. Data are median for continuous variables and absolute numbers for categorical variables

Supplementary Material 8. Relationship between the length of the first cycle in prone position and the arterial oxygenation response to the first cycle of prone positioning evaluated at the end of prone position beforeand after re-supination. The x-axis shows the predictor and the y-axis shows the effect of the predictor on the outcome. The solid line represents the regression line estimated by the logistic regression model, and the gray bands show the confidence intervals. When the relationship was found to be non-linear, the change-point was identified. Abbreviations. PaO_2_/FiO_2_ arterial partial pressure of oxygen to inspire fraction of oxygen ratio. OR, odds ratio. 95%CI, 95% confidence interval.

Supplementary Material 9. Subgroup analysis on ICU mortality according to recruitment type

## Data Availability

Individual participant data that underlie the results reported in this article, after de-identification, data dictionary, study protocol, statistical analysis plan, informed consent form, and analytic code will be available to any researchers who provide a methodologically sound proposal, immediately following publication and without end date. Proposals should be directed to the corresponding author. To gain access, data requestors will need to sign a data access agreement.

## Abbreviations

ARDS: Acute respiratory distress syndrome (ARDS)
COVID-19: Coronavirus disease 2019
V-V ECMO: Veno-venous extracorporeal membrane oxygenation
ICU: Intensive care unit
PaO2/FiO2: Arterial partial pressure of oxygen to fraction of inspired oxygen ratio
Crs: Static compliance of the respiratory system
Delta-PP: Difference in prone position
Delta-PostPP: Difference after prone positioning
