# Supplementary material for: Oxygenation improvement and duration of prone positioning are associated with ICU mortality in mechanically ventilated COVID-19 patients
Source: Ann Intensive Care. 2025 Jan 28;15:20. doi: 10.1186/s13613-025-01438-y (PMC11775368; doi:10.1186/s13613-025-01438-y)
Supplement: Supplementary file 2 — Supplementary Material 2. Extended Methods [file 13613_2025_1438_MOESM2_ESM.docx]

**TITLE:** Oxygenation Improvement And Duration Of Prone Positioning Are Associated With ICU Mortality In Mechanically Ventilated COVID-19 Patients.

**AUTHORS:**

Silvia De Rosa, Nicolò Sella, Giacomo Bellani, Giuseppe Foti, Andrea Cortegiani, Giulia Lorenzoni, Dario Gregori, Annalisa Boscolo, Lucia Cattin, Muhammed Elhadi, Giorgio Fullin, Eugenio Garofalo, Leonardo Gottin, Alberto Grassetto, Salvatore Maurizio Maggiore, Elena Momesso, Mario Peta, Daniele Poole, Roberto Rona, Ivo Tiberio, Andrea Zanoletti, Emanuele Rezoagli, Paolo Navalesi, for the SIAARTI Study Group.

**ONLINE DATA SUPPLEMENT**

**Extended Methods**

**Study design**

The PROVENT-C19 is a web-based, multicenter, observational registry originally developed by the COVID-19 Veneto Intensive Care Unit (ICU) Network research group [E1,E2], then implemented nationally with the support of the Italian Society of Anesthesia, Analgesia, Resuscitation and Intensive Care (SIAARTI), and spread internationally with the endorsement of the European Society of Intensive Care Medicine (ESICM), and the European Society of Anaesthesiology and Intensive Care (ESAIC) [E1]. There was no funding source for this study.

The registry was designed following the Declaration of Helsinki and the study protocol was firstly approved by the Ethics Committee of the Saint Bortolo Hospital, Vicenza, Italy (Study ID Numbers: 22/21). Data were collected through the workflow methodology and software solution of research electronic data capture (REDCap): for every participating center, a local principal investigator was identified, who received a personal username and password alongside with the instructions on how to use the electronic collection form. Each patient was identified through a patient identification number and all personal information was processed with pseudonymization in compliance with the European Union General Data Protection Regulation. Patient consent was obtained according to the national regulations of each participating Institution. In cases the patient was incompetent because of critical illness or the use of sedative or anesthetic drugs, consent could be delayed, and a provision for delayed consent was applied: as soon as competent, each patient was fully informed on what had been done, and a written permission of using data collected was obtained [E3]. The patients or their legal surrogates were informed of their right to request that the study procedures be discontinued and their right to refuse the study-related use of their medical records [E3]. The Strengthening the Reporting of Observational studies in Epidemiology (STROBE) reporting guideline checklist for observational studies was used for reporting this study (**Online Data Supplement - Table E1**).

**Patients**

The PROVENT-C19 Registry included, either prospectively or retrospectively, consecutive adult patients with laboratory-confirmed COVID-19 infection who underwent invasive mechanical ventilation due to COVID-19 related acute respiratory failure and were treated with prone positioning from December 31^st^ 2019 to January 1^st^ 2023. Patients were excluded if they refused the consent to participate or if they presented contraindications to prone position, i.e. unstable spinal, pelvic or long bone fractures, severe hemodynamic instability, open abdominal wounds, and late-term pregnancy [E4,E5].

**Procedures**

The following data were collected and stored for analysis: *i)* day and hour of both hospital and ICU admission; *ii)* demographics data; *iii)* comorbidities; *iv)* respiratory support before tracheal intubation; *v)* clinical and laboratory parameters (i.e., Sequential Organ Failure Assessment Score, Glasgow Coma Score, serum C-reactive protein, serum procalcitonin, serum D-Dimer concentration) at ICU admission; *vi)* arterial blood gases at ICU admission and before intubation; *vii)* total number and length of prone position cycles with invasive mechanical ventilation during ICU stay. In particular, for each cycle in prone position, ventilator settings (i.e., tidal volume [Vt] to predicted body weight, respiratory rate, positive end-expiratory pressure [PEEP], fraction of inspired oxygen [FiO_2_]), respiratory mechanics parameters (i.e., driving pressure [DP], static compliance of the respiratory system [Crs]) [E6], ventilatory ratio [E7], and arterial blood gases values were recorded at different time points (**Figure 1**). The following parameters were also registered: *i)* ICU and hospital survival and length of stay; *ii)* incidence and outcome of extubation; *iii)* 28-day ventilator free days [E8]; *iv)* need for inhaled nitric oxide (iNO), tracheostomy, continuous renal replacement therapy (CRRT), ECMO or extracorporeal carbon dioxide removal (ECCO2R); *v)* complications related to prone positioning (i.e., pressure ulcers, endotracheal tube obstruction or accidental removal, vascular lines or chest tube dislodgement, severe hemodynamic instability, acute hemorrhage) [E4].

**Outcomes**

Physiological response to prone position was evaluated at different time points (**Figure 1**), calculating for arterial partial pressure of oxygen to FiO_2_ ratio (PaO_2_/FiO_2_), ventilatory ratio, (as proxy for dead space) [E9] and Crs:

- Difference in prone position (Delta-PP): the difference between the last available values within the last 30 minutes in prone and supine position (prior to being turned prone), respectively;
- Difference after prone positioning (Delta-PostPP): the difference between the first available value within the first 30 minutes in supine position after the end of the prone position cycle and the last available value in supine position before being turned prone (in the last 30 minutes prior to being turned prone).

Patients were categorized according to:

- ICU survival (i.e., survivors versus non survivors);
- length of the first cycle of prone position in three groups: 1) short pronation (i.e., <16 hours); 2) long pronation (between 16 and 24 hours); and 3) extended pronation (i.e., >24 hours).

**Data management**

We checked for quality and integrity of the reported data. Missing, extreme or implausible values were returned to the local principal investigator for review and correction. The remaining missing and questionable data (e.g. variables registered more than 30 minutes later than required by the study design) were omitted from the analyses. Complete case analysis was performed.

**Statistical Analysis**

Categorical data are presented as absolute numbers (n) and percentages (%). For continuous data medians and I and III quartiles are used. The distribution of categorical variables was compared with the Pearson's Chi-squared test or the Fisher's exact test, whichever appropriate, while the distribution of continuous variables was compared using Wilcoxon rank sum test. To account for multiplicity of testing, Benjamini-Hochberg correction was employed and *q-value* (i.e., the *p-value* adjusted for false discovery rate that helps maintain a balance between discovering statistically significant results and controlling for the rate of type I errors) was presented.

Univariable logistic regression models were employed to assess the association between patients' characteristics or physiological response to prone position and ICU mortality. Results were reported as Odds Ratio (OR), 95% Confidence Interval (CI), and *p-value*. For continuous predictors, the OR was presented on the interquartile range of the variable. If the association was nonlinear, restricted cubic splines were used to estimate the models and the change-point was identified. To account for clustering effect within the same center, the Huber-White method was employed to adjust the variance-covariance matrix.

A multivariable logistic regression model was used to evaluate the association between physiological response to prone position and ICU mortality after adjusting for relevant confounders. The tested variables were selected for their recognized clinical value by the study steering committee (“educated guess”) [E10] and included: age at hospital admission, arterial hypertension, chronic heart failure, D-dimer and SOFA score at ICU admission, length of the first cycle of prone positioning, Delta-PP and Delta-PostPP for PaO_2_/FiO_2_, Delta-PP and Delta-PostPP for ventilatory ratio, and Delta-PP and Delta-PostPP for Crs. Delta-PP and Delta-PostPP for Crs were censored because missing data exceeded 20% of the overall population. Additionally, Delta-PP and Delta-PostPP either for PaO_2_/FiO_2_, or ventilatory ratio were tested in two different models because of multicollinearity [E6]. The multivariable analysis was corrected to account for the potential confounding effect of the ICU admission period.

All statistical tests were 2-tailed, and statistical significance was defined as *p* < 0.05. All analyses have been conducted using R version 4.0.3 (R foundation for Statistical Computing, Vienna, Austria).

**E-REFERENCES**

E1  De Rosa S, Sella N, Rezoagli E, et al. The PROVENT-C19 registry: A study protocol for international multicenter SIAARTI registry on the use of prone positioning in mechanically ventilated patients with COVID-19 ARDS. PLoS One. 2022 Dec 30;17(12):e0276261.

E2  Pasin L, Sella N, Correale C, et al. Regional COVID-19 Network for Coordination of SARS-CoV-2 outbreak in Veneto, Italy. J Cardiothorac Vasc Anesth. 2020 Sep;34(9):2341-2345.

E3  Gattinoni L, Caironi P, Cressoni M, et al. Lung recruitment in patients with the acute respiratory distress syndrome. N Engl J Med. 2006 Apr 27;354(17):1775-86.

E4  Guérin C, Albert RK, Beitler J, et al. Prone position in ARDS patients: why, when, how and for whom. Intensive Care Med. 2020 Dec;46(12):2385-2396.

E5 Zarantonello F, Sella N, Pettenuzzo T, et al. Early Physiologic Effects of Prone Positioning in COVID-19 Acute Respiratory Distress Syndrome. Anesthesiology. 2022 Sep 1;137(3):327-339.

E6 Boscolo A, Sella N, Lorenzoni G, et al. Static compliance and driving pressure are associated with ICU mortality in intubated COVID-19 ARDS. Crit Care. 2021 Jul 28;25(1):263.

E7 Sinha P, Calfee CS, Beitler JR, et al. Physiologic Analysis and Clinical Performance of the Ventilatory Ratio in Acute Respiratory Distress Syndrome. Am J Respir Crit Care Med. 2019 Feb 1;199(3):333-341.

E8 Yehya N, Harhay MO, Curley MAQ, Schoenfeld DA, Reeder RW. Reappraisal of Ventilator-Free Days in Critical Care Research. Am J Respir Crit Care Med. 2019 Oct 1;200(7):828-836.

E9 Zheng M. Dead space ventilation-related indices: bedside tools to evaluate the ventilation and perfusion relationship in patients with acute respiratory distress syndrome. Crit Care. 2023 Feb 3;27(1):46.

E10 Moons KG, Altman DG, Reitsma JB, Ioannidis JP, Macaskill P, Steyerberg EW, et al. Transparent Reporting of a multivariable prediction model for Individual Prognosis or Diagnosis (TRIPOD): explanation and elaboration. Annals of internal medicine. 2015;162(1):W1–73.
