# Supplementary figures and images for: Oxygenation improvement and duration of prone positioning are associated with ICU mortality in mechanically ventilated COVID-19 patients
Source: Ann Intensive Care. 2025 Jan 28;15:20. doi: 10.1186/s13613-025-01438-y (PMC11775368; doi:10.1186/s13613-025-01438-y)

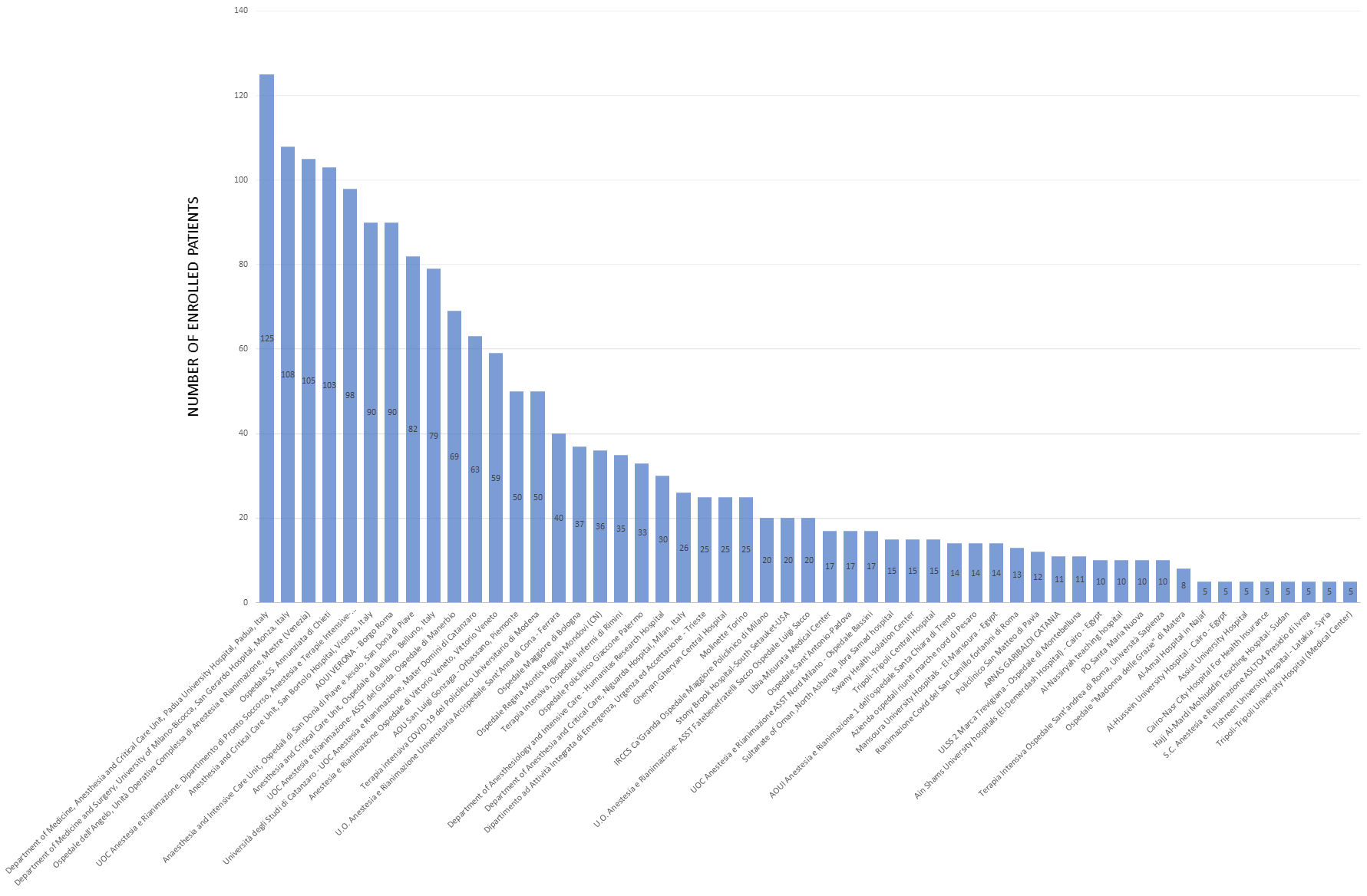

Supplement: Supplementary file 3 — Supplementary Material 3. Patients enrollment among the participating centers [file 13613_2025_1438_MOESM3_ESM.tif]

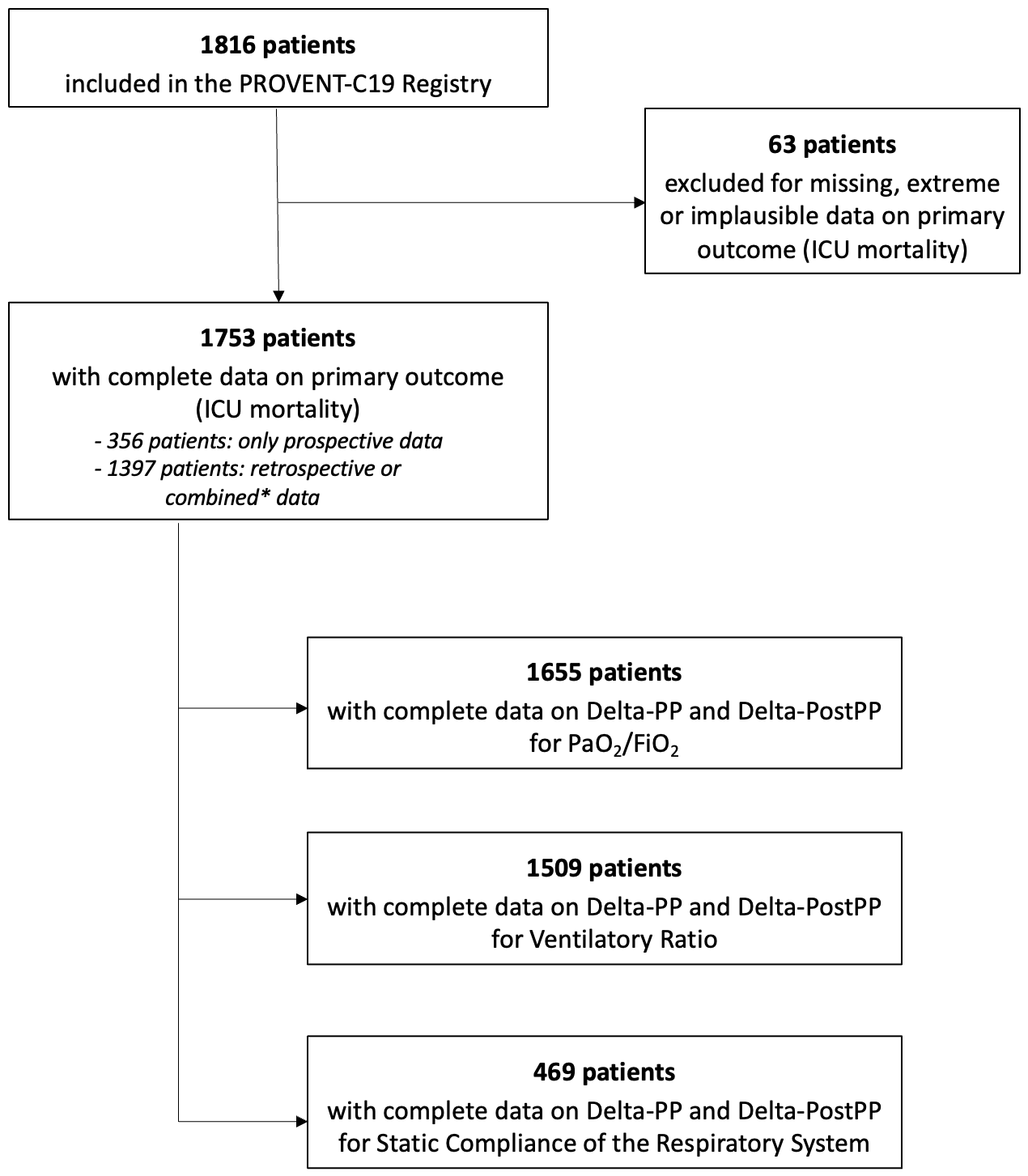

Supplement: Supplementary file 4 — Supplementary Material 4. Study patients flowchart. Abbreviations. ICU, intensive care unit. PaO2/FiO2 arterial partial pressure of oxygen to inspire fraction of oxygen ratio. *Combined enrollment indicates patients who were included in the registry after the end of the first cycle of prone position, but before hospital discharge, thus part of the data has been collected retrospectively, while other information has been registered prospectively. [file 13613_2025_1438_MOESM4_ESM.tiff]

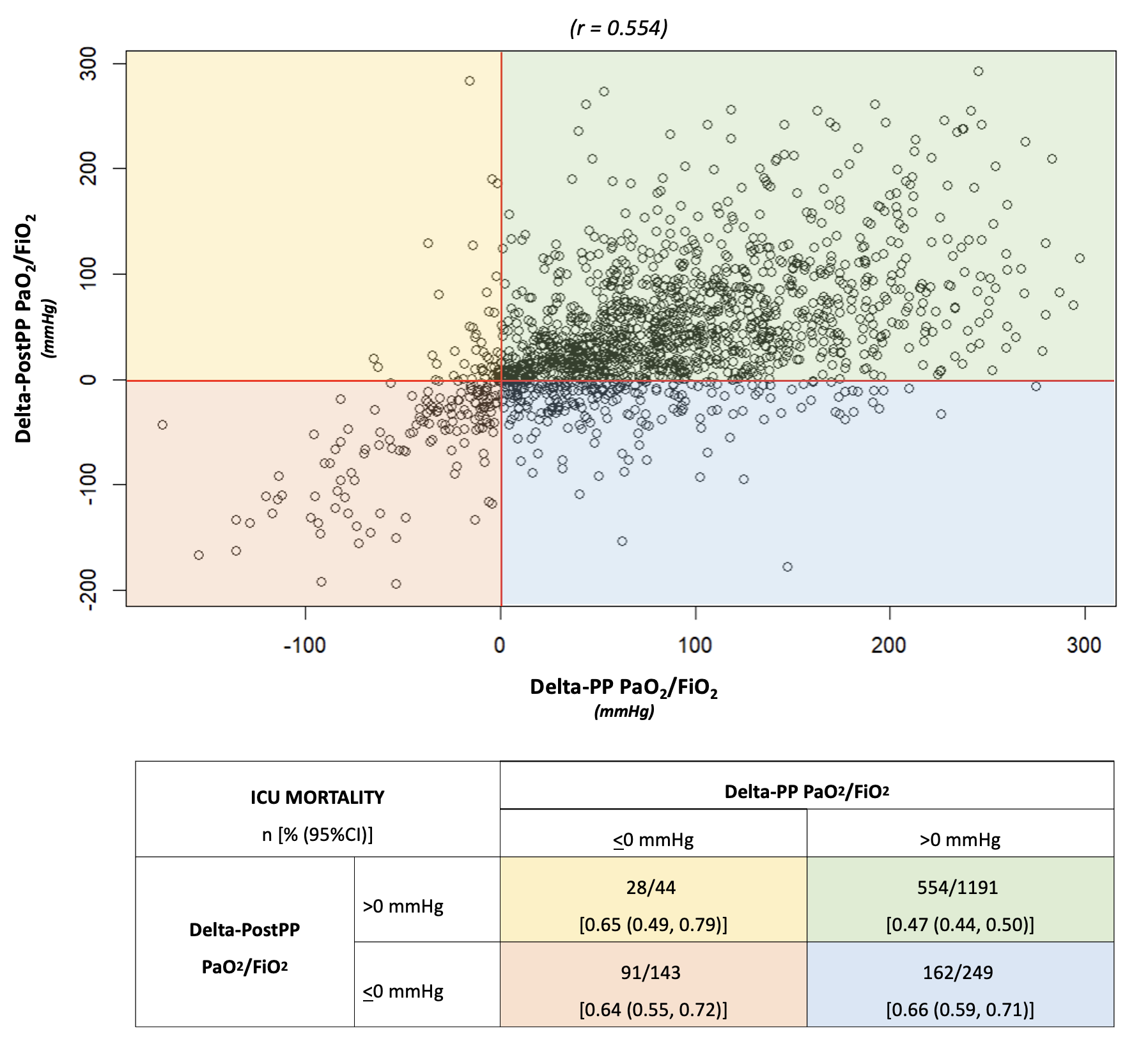

Supplement: Supplementary file 6 — Supplementary Material 6. Relationship between the arterial oxygenation response to the first cycle of prone positioning evaluated at the end of prone position before and after being turned supine. Abbreviations. PaO2/FiO2 arterial partial pressure of oxygen to inspire fraction of oxygen ratio. 95%CI, 95% confidence interval. ICU, intensive care unit. [file 13613_2025_1438_MOESM6_ESM.tiff]

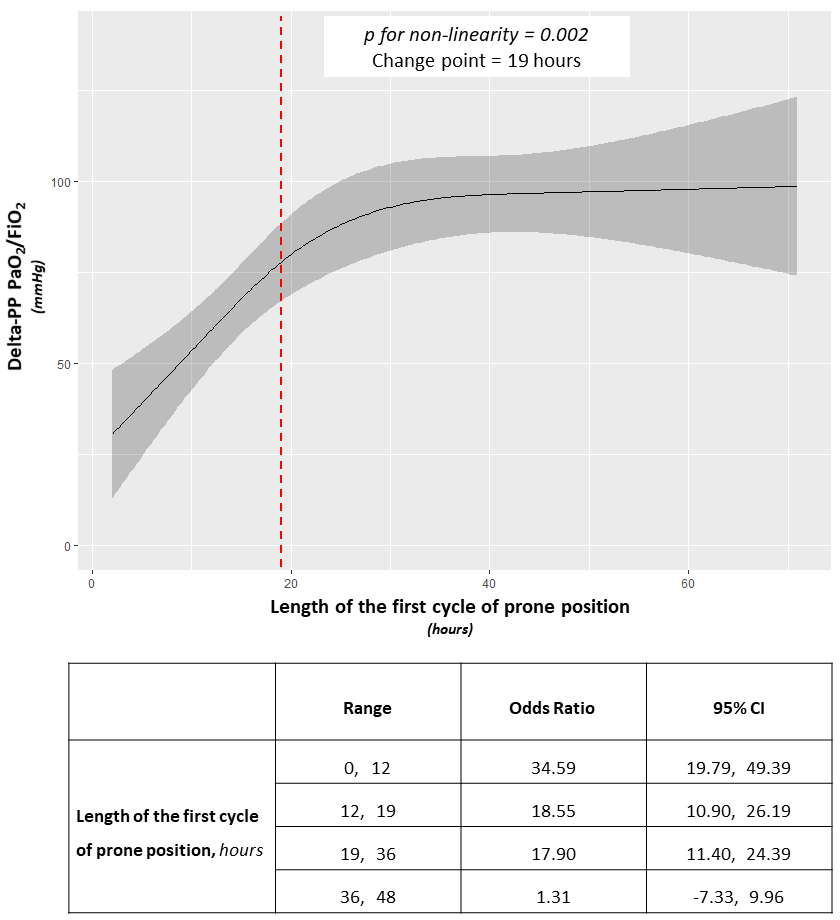

Supplement: Supplementary file 8 — Supplementary Material 8. Relationship between the length of the first cycle in prone position and the arterial oxygenation response to the first cycle of prone positioning evaluated at the end of prone position beforeand after re-supination. The x-axis shows the predictor and the y-axis shows the effect of the predictor on the outcome. The solid line represents the regression line estimated by the logistic regression model, and the gray bands show the confidence intervals. When the relationship was found to be non-linear, the change-point was identified. Abbreviations. PaO2/FiO2 arterial partial pressure of oxygen to inspire fraction of oxygen ratio. OR, odds ratio. 95%CI, 95% confidence interval. [file 13613_2025_1438_MOESM8_ESM.tif]
