# Supplementary material for: Oxygenation improvement and duration of prone positioning are associated with ICU mortality in mechanically ventilated COVID-19 patients
Source: Ann Intensive Care. 2025 Jan 28;15:20. doi: 10.1186/s13613-025-01438-y (PMC11775368; doi:10.1186/s13613-025-01438-y)
Supplement: Supplementary file 5 — Supplementary Material 5. Main clinical characteristics of the study population. Data are median for continuous variables and absolute numbers for categorical variables [file 13613_2025_1438_MOESM5_ESM.docx]

**TITLE:** Oxygenation Improvement And Duration Of Prone Positioning Are Associated With ICU Mortality In Mechanically Ventilated COVID-19 Patients.

**AUTHORS:**

Silvia De Rosa, Nicolò Sella, Giacomo Bellani, Giuseppe Foti, Andrea Cortegiani, Giulia Lorenzoni, Dario Gregori, Annalisa Boscolo, Lucia Cattin, Muhammed Elhadi, Giorgio Fullin, Eugenio Garofalo, Leonardo Gottin, Alberto Grassetto, Salvatore Maurizio Maggiore, Elena Momesso, Mario Peta, Daniele Poole, Roberto Rona, Ivo Tiberio, Andrea Zanoletti, Emanuele Rezoagli, Paolo Navalesi, for the SIAARTI Study Group.

**ONLINE DATA SUPPLEMENT TABLE E2. Demographic and baseline characteristics of the overall study population, including patients with incomplete data on primary outcome.**

Data are median (I quartile-III quartile) for continuous variables and absolute numbers (percentages) for categorical variables.

| **CHARACTERISTICS** | **OVERALL** | |
| --- | --- | --- |
|  | **N** |  |
| Demographics  Age, *years*  Male, *n (%)*  BMI, *kg/m^2^*  Pandemic wave, *n (%)*:  -1^st^ wave (January 2020 - August 2020)  -2^nd^ wave (September 2020 - March 2021)  -3^rd^ wave (April 2021 - December 2022) | 1803  1811  1719  1804 | 66 (57, 73)  1278 (71.0)  28 (25, 32)  170 (9.0)  841 (47.0)  793 (44.0) |
| Comorbidities  COPD, *n(%)*  Arterial hypertension, *n (%)*  Chronic heart failure, *n (%)*  Cerebral vasculopathy, *n (%)*  Diabetes mellitus, *n (%)*  Chronic kidney disease, *n (%)*  Home renal replacement therapy, *n (%)*  Chronic liver failure, *n (%)*  Cancer, *n (%)*  Immunological deficiency, *n (%)* | 1799  1800  1801  1800  1801  1800  1800  1800  1800  1799 | 170 (9.4)  998 (55.0)  241 (13.0)  73 (4.1)  457 (25.0)  79 (4.4)  16 (0.9)  34 (1.9)  79 (4.4)  134 (7.4) |
| Before ICU admission  COVID-19 vaccination, *n (%)*  Hospitalization before ICU admission, *days*  Corticosteroids before ICU admission, *n(%)*  Anticoagulant therapy before ICU admission, *n (%)*  Non-invasive respiratory support before IMV, *n (%)*  Non-invasive respiratory support before IMV, *days* | 1265  1803  1779  1779  1798  1363 | 189 (15.0)  3 (1, 6)  1244 (70.0)  1176 (66.0)  1563 (87.0)  3 (1, 5) |
| ICU admission  IMV at ICU admission, *n (%)*  PaO_2_/FiO_2_ at ICU admission, *mmHg*  Glasgow Coma Scale at ICU admission  SOFA at ICU admission  White Blood Cells at ICU admission, *x10^9^/L*  CRP at ICU admission, *mg/L*  Procalcitonin at ICU admission, *mcg/L*  D-Dimer at ICU admission, *mcg/L* | 1797  1777  1744  1753  1769  1447  1273  1204 | 532 (30.0)  87 (67, 117)  15 (15, 15)  4 (3, 6)  10 (7, 14)  43 (12, 130)  0.23 (0.10, 0.63)  984 (285, 2737) |

**Abbreviations.** ICU, intensive care unit. OR, Odds Ratio. 95%CI, 95% Confidence Interval. BMI, body mass index. COPD, chronic obstructive pulmonary disease. IMV, invasive mechanical ventilation. PaO_2_/FiO_2_ arterial partial pressure of oxygen to inspire fraction of oxygen ratio. SOFA, sequential organ failure assessment. CRP, C-reactive protein.
