# Supplementary material for: Oxygenation improvement and duration of prone positioning are associated with ICU mortality in mechanically ventilated COVID-19 patients
Source: Ann Intensive Care. 2025 Jan 28;15:20. doi: 10.1186/s13613-025-01438-y (PMC11775368; doi:10.1186/s13613-025-01438-y)
Supplement: Supplementary file 7 — Supplementary Material 7. Baseline characteristics and main clinical outcomes of the study population stratified according to the length of the first cycle in prone position. Data are median for continuous variables and absolute numbers for categorical variables [file 13613_2025_1438_MOESM7_ESM.docx]

**TITLE:** Oxygenation Improvement And Duration Of Prone Positioning Are Associated With ICU Mortality In Mechanically Ventilated COVID-19 Patients.

**AUTHORS:**

Silvia De Rosa, Nicolò Sella, Giacomo Bellani, Giuseppe Foti, Andrea Cortegiani, Giulia Lorenzoni, Dario Gregori, Annalisa Boscolo, Lucia Cattin, Muhammed Elhadi, Giorgio Fullin, Eugenio Garofalo, Leonardo Gottin, Alberto Grassetto, Salvatore Maurizio Maggiore, Elena Momesso, Mario Peta, Daniele Poole, Roberto Rona, Ivo Tiberio, Andrea Zanoletti, Emanuele Rezoagli, Paolo Navalesi, for the SIAARTI Study Group.

**ONLINE DATA SUPPLEMENT TABLE E2.** Baseline characteristics and main clinical outcomes of the study population stratified according to the length of the first cycle in prone position**.**

Data are median (I quartile-III quartile) for continuous variables and absolute numbers (percentages) for categorical variables.

| **CHARACTERISTICS** | **N**  **n = 1673** | **SHORT PRONATION**  **(<16 hours)**  **n = 424 (25.3%)** | **LONG PRONATION**  **(16-24 hours)**  **n = 807 (48.2%)** | **EXTENDED PRONATION**  **(>24 hours)**  **n = 442 (26.5%)** | **Q-VALUE** |
| --- | --- | --- | --- | --- | --- |
| Demographics  Age, *years*  Male, *n (%)*  BMI, *kg/m^2^*  Pandemic wave, *n (%)*:  -1^st^ wave (January 2020 - August 2020)  -2^nd^ wave (September 2020 - March 2021)  -3^rd^ wave (April 2021 - December 2022) | 1672  1670  1590  1673 | 66 (56, 72)  298 (70.0)  27.8 (24.5, 31.7)  *#  68 (16.0)  177 (42.0)  179 (42.0) | 66 (58, 73)  574 (71.0)  27.8 (25.4, 32.6)  *^  79 (10.0)  431 (53.0)  297 (37.0) | 65 (57, 72)  311 (71.0)  28.9 (25.8, 32.4)  #^  9 (2.0)  203 (46.0)  230 (52.0) | 0.300  >0.900  0.200  **<0.001** |
| Comorbidities  COPD, *n(%)*  Arterial hypertension, *n (%)*  Chronic heart failure, *n (%)*  Cerebral vasculopathy, *n (%)*  Diabetes mellitus, *n (%)*  Chronic kidney disease, *n (%)*  Home renal replacement therapy, *n (%)*  Chronic liver failure, *n (%)*  Cancer, *n (%)*  Immunological deficiency, *n (%)* | 1670  1671  1672  1671  1672  1671  1671  1671  1671  1670 | 42 (9.9)  238 (56.0)  79 (19.0)*  24 (5.7)  112 (26.0)  27 (6.4)*#  9 (2.1)  9 (2.1)  17 (4.0)  42 (9.9) | 86 (11.0)  468 (58.0)  104 (13.0)*  30 (3.7)  196 (24.0)  33 (4.1)*  4 (0.5)  18 (2.2)  32 (4.0)  56 (7.0) | 32 (7.2)  227 (51.0)  41(9.3)  13 (2.9)  108 (24.0)  13 (2.9)#  1 (6.7)  2 (0.5)  24 (5.4)  29 (6.6) | 0.200  0.130  **<0.001**  0.200  0.800  0.082  0.130  0.100  0.600  0.200 |
| Before ICU admission  COVID-19 vaccination, *n (%)*  Hospitalization before ICU admission, *days*  Corticosteroids before ICU admission, *n(%)*  Anticoagulant therapy before ICU admission, *n (%)*  Non-invasive respiratory support before IMV, *n (%)*  Non-invasive respiratory support before IMV, *days* | 1162  1672  1666  1666  1670  1295 | 51 (16.0)*  3 (1, 6)  273 (65.0)#  258 (61.0)#  348 (82.0)*  3 (1, 5) | 54 (9.8)*^  3 (1, 7)  546 (68.0)^  518 (65.0)^  723 (90.0)*  3 (1, 6) | 54 (18.0)^  3 (1, 6)  355 (80.0)#^  337 (76.0)#^  393 (89.0)  3 (1, 5) | **0.003**  0.300  **<0.001**  **<0.001**  **0.001**  0.700 |
| ICU admission  IMV at ICU admission, *n (%)*  PaO_2_/FiO_2_ at ICU admission, *mmHg*  Glasgow CS at ICU admission  SOFA at ICU admission  White Blood Cells at ICU admission, *x10^9^/L*  CRP at ICU admission, *mg/L*  Procalcitonin at ICU admission, *mcg/L*  D-Dimer at ICU admission, *mcg/L* | 1673  1662  1638  1652  1656  1358  1201  1131 | 127 (30.0)  86 (67, 114)  15 (14, 15)*#  4 (4, 7)*#  11 (7, 15)  51 (12, 142)#  0.25 (0.10, 0.67)#  1059 (327, 4524) | 254 (31.0)  89 (68, 119)  15 (15, 15)*  4 (3, 5)*  10 (7, 14)  47 (12, 128)^  0.24 (0.11, 0.70)^  928 (222, 2105) | 122 (28.0)  87 (69, 117)  15 (15, 15)#  4 (3, 5)#  10 (7, 14)  26 (11, 115)#^  0.20 (0.10, 0.47)#^  964 (323, 2550) | 0.400  0.300  **<0.001**  **<0.001**  0.500  **0.024**  **0.034**  0.075 |
| ICU, before the first cycle of prone position  Time in supine position, *hours*  Neuro-muscolar blocking drugs, *n (%)*  Renal RT, *n (%)*  ECMO or ECCO_2_R, *n (%)*  iNO, *n (%)*  VT, *mL/kg IBW*  Respiratory rate, *bpm*  PEEP, *cmH_2_O*  Pplat, *cmH_2_O*  Driving pressure, *cmH_2_O*  Crs, *mL/cmH_2_O*  FiO_2_  PaO_2_/FiO_2_, *mmHg*  pH  PaCO_2_, *mmHg* | 1637  1669  1249  1666  1645  1498  1590  1607  638  619  558  1645  1635  1633  1637 | 8 (3, 30)#  227 (54.0)*#  15 (5.0)*#  12 (2.8)#  9 (2.2)  6.96 (5.99, 7.85)  20 (16, 24)  10 (8, 12)  23 (19, 26)  12 (9, 13)  38 (30, 50)  0.9 (0.7, 1.0)#  93 (68, 120)  7.37 (7.30, 7.44)*  46 (39, 54) | 6 (2, 26)^  553 (69.0)*  8 (1.4)*  12 (1.5)  20 (2.5)  6.74 (6.02, 7.51)  20 (18, 25)  10 (8, 12)  23 (21, 26)  12 (10, 14)  40 (31, 50)  0.9 (0.7, 1.0)^  91 (70, 123)^  7.39 (7.32, 7.44)*^  47 (40, 54) | 3 (1, 10)#^  292 (66.0)#  5 (1.4)#  5 (1.1)#  9 (2.1)  6.82 (6.18, 7.63)  20 (18, 24)  10 (8, 12)  23 (20, 26)  12 (10, 14)  37 (31, 45)  1.0 (0.8, 1.0)#^  82 (65, 115)^  7.40 ( 7.33, 7.45)^  45 (38, 53) | **<0.001**  **<0.001**  **0.002**  **0.014**  >0.900  0.300  0.200  0.710  0.700  0.900  0.400  **<0.001**  **0.005**  **<0.001**  0.300 |
| Prone position (last values during the cycle)  Time in prone position, *hours*  Neuro-muscolar blocking drugs, *n (%)*  ECMO or ECCO_2_R, *n (%)*  iNO, *n (%)*  VT*, mL/kg IBW*  Respiratory rate, *bpm*  PEEP, *cmH_2_O*  Pplat, *cmH_2_O*  Driving pressure, *cmH_2_O*  Crs, *mL/cmH_2_O*  FiO_2_  PaO_2_/FiO_2_, *mmHg*  pH  PaCO_2_*, mmHg*  Pressure ulcers, *n (%)* | 1673  1669  1664  1630  1509  1583  1594  744  717  657  1630  1626  1627  1629  1649 | 13 (10, 15)*#  383 (90.0)*#  12 (2.8)  7 (1.7)  6.92 (6.06, 7.79)  20 (16, 24)  10 (8, 12)  23 (20, 26)  11 (10, 13)  38 (30, 47)  0.7 (0.6, 0.9)*#  146 (102, 205)*#  7.37 (7.32, 7.42)*#  48 (40, 56)  90 (22.0) | 18 (17, 21)*^  771 (96.0)*  12 (1.5)  29 (3.7)  6.67 (6.02, 7.48)  22 (18, 25)  10 (9, 12)  23 (20, 25)  11 (9, 13)  40 (32, 50)  0.7 (0.5, 0.8)*^  172 (128, 234)*  7.40 (7.35, 7.45)*  47 (41, 55)  223 (28.0) | 40 (32, 48)#^  430 (98.0)#  8 (1.8)  12 (2.7)  6.82 (6.06, 7.67)  22 (18, 25)  10 (8, 12)  22 (20, 25)  11 (9, 13)  41 (33, 50)  0.6 (0.4, 0.7)#^  178 (127, 240)#  7.42 (7.38, 7.45)#  48 (42, 55)  120 (27.0) | **<0.001**  **<0.001**  0.400  0.300  0.300  0.069  0.300  0.100  0.300  0.200  **<0.001**  **<0.001**  **<0.001**  0.300  0.120 |
| Delta PP  PaO_2_/FiO_2_*, mmHg*  Ventilatory ratio  Crs, *mL/cmH_2_O*  Dynamic Crs*, mL/cmH_2_O*  Delta APP  PaO_2_/FiO_2_*, mmHg*  Ventilatory ratio  Crs*, mL/cmH_2_O*  Dynamic Crs*, mL/cmH_2_O* | 1589  1444  439  311  1584  1405  368  282 | 45 (6, 95)*#  0.03 (-0.15, 0.24)  0 (-5, 5)  0 (-4, 5)  17 (-1, 58)*#  0.06 (-0.16, 0.34)  1 (-3, 8)  1 (-5, 5) | 73 (33, 125)*  0.01 (-0.16, 0.25)  1 (-2, 6)  0 (-3, 4)  28 (-1, 66)*^  0.08 (-0.16, 0.33)  0 (-5, 6)  0 (-3, 6) | 85 (37, 136)#  0.09 (-0.15, 0.36)  2 (-4, 10)  0 (-3, 4)  43 (10, 90)#^  0.13 (-0.14, 0.42)  0 (-5, 7)  -2 (-4, 2) | **<0.001**  0.067  0.150  >0.900  **<0.001**  0.400  0.700  0.092 |
| Outcome  Discharged alive from ICU, *n (%)*  Discharged alive from hospital, *n (%)*  ICU LOS, *days*  Hospital LOS, *days*  28-day ventilator free days  Tracheostomy, *n (%)*  Renal replacement therapy, *n (%)*  ECMO or ECCO_2_R, *n (%)*  iNO, *n (%)*  Cycles of prone position, *n (%)*  Overall time in prone position, *hours* | 1651  1620  1647  1612  943  1651  1644  1444  1576  1673  1443 | 184 (44.0)#  167 (41.0)#  13 (8, 23)*#  22 (13, 38)*#  0 (0, 0)  101 (24.0)  68 (16.0)*#  16 (3.8)*#  19 (5.0)  3 (2, 5)  42 (25, 74)*# | 336 (42.0)^  312 (40.0)^  16 (10, 27)*  25 (16, 40)*  0 (0, 0)  215 (27.0)  78 (9.8)*  11 (1.4)*  50 (6.5)  3 (2, 5)^  59 (39, 87)*^ | 256 (58.0)#^  243 (56.0)#^  15 (9, 25)#  27 (17, 42)#  0 (0, 0)  123 (28.0)  33 (7.6)#  5 (1.1)#  25 (5.9)  2 (2, 4)^  89 (63, 135)#^ | **<0.001**  **<0.001**  **<0.001**  **0.002**  0.200  0.500  **<0.001**  **0.014**  0.700  **<0.001**  **<0.001** |

***short vs long pronation: q <0.05**

**#short vs extended pronation: q <0.05**

**^long vs extended: q <0.05**

**Abbreviations.** BMI, body mass index. COPD, chronic obstructive pulmonary disease. ICU, intensive care unit. IMV, invasive mechanical ventilation. PaO_2_/FiO_2_ arterial partial pressure of oxygen to inspire fraction of oxygen ratio. SOFA, sequential organ failure assessment. CRP, C-reactive protein. Renal RT, renal replacement therapy. ECMO, extracorporeal membrane oxygenation. ECCO_2_R, extracorporeal carbon dioxide removal. iNO, inhale nitric oxide. VT, tidal volume. IBW, ideal body weight. PEEP, positive end-expiratory pressure. Pplat, plateau pressure. Crs, static compliance of the respiratory system. PaCO_2_ arterial partial pressure of carbon dioxide. LOS, length of stay.
