# Supplementary material for: Oxygenation improvement and duration of prone positioning are associated with ICU mortality in mechanically ventilated COVID-19 patients
Source: Ann Intensive Care. 2025 Jan 28;15:20. doi: 10.1186/s13613-025-01438-y (PMC11775368; doi:10.1186/s13613-025-01438-y)
Supplement: Supplementary file 9 — Supplementary Material 9. Subgroup analysis on ICU mortality according to recruitment type [file 13613_2025_1438_MOESM9_ESM.docx]

**TITLE:** Oxygenation Improvement And Duration Of Prone Positioning Are Associated With ICU Mortality In Mechanically Ventilated COVID-19 Patients.

**AUTHORS:**

Silvia De Rosa, Nicolò Sella, Giacomo Bellani, Giuseppe Foti, Andrea Cortegiani, Giulia Lorenzoni, Dario Gregori, Annalisa Boscolo, Lucia Cattin, Muhammed Elhadi, Giorgio Fullin, Eugenio Garofalo, Leonardo Gottin, Alberto Grassetto, Salvatore Maurizio Maggiore, Elena Momesso, Mario Peta, Daniele Poole, Roberto Rona, Ivo Tiberio, Andrea Zanoletti, Emanuele Rezoagli, Paolo Navalesi, for the SIAARTI Study Group.

**ONLINE DATA SUPPLEMENT TABLE E4.** Subgroup analysis on ICU mortality according to recruitment type (Prospective vs Retrospective)**.**

|  | **PaO_2_/FiO_2_** | **Ventilatory Ratio** | **Crs** |
| --- | --- | --- | --- |
| **Delta-PP** | P-value for interaction term: 0.720 | P-value for interaction term (linear): 0.327  P-value for interaction term (non-linear): 0.087 | P-value for interaction term: 0.809 |
| **Delta-PostPP** | P-value for interaction term: 0.772 | P-value for interaction term (linear): 0.668  P-value for interaction term (non-linear): 0.881 | P-value for interaction term: 0.368 |

**Abbreviations.** ICU, intensive care unit. Crs, static compliance of the respiratory system. PaO_2_/FiO_2_ arterial partial pressure of oxygen to inspire fraction of oxygen ratio.
